# The effects of scoliosis bracing on parent and child perception of adolescent’s quality of life

**DOI:** 10.1186/1748-7161-8-S1-O56

**Published:** 2013-06-03

**Authors:** P Knott, C Anderson, M Werner, H Wilson, D Speers

**Affiliations:** 1 Rosalind Franklin University of Medicine, Chicago, USA

## Background

Improvement of quality of life is the most important reason for treating AIS, yet research on this topic is very limited with less than 2% of research on scoliosis including a QoL measurement [1]. The BrQ is a scoliosis quality of life survey that has been shown to be reliable, valid, and responsive to change in adolescents with AIS who are treated conservatively with bracing [2]. Although many studies have shown that adolescents with scoliosis have QoL indicators that are lower than their non-scoliosis counterparts, the question arises as to whether their feelings may be intensified and exaggerated during their adolescent years. One way to assess this would be to compare the adolescent assessment to the assessment by the parent. This has been done once using the SRS questionnaire in surgical patients, and discrepancies were found between parent and child [3]. This study gave the BrQ survey to adolescent patients who were wearing a brace and their parents to look at the correlation between the scores.

## Aim

The goal of this study was to compare the BrQ results of adolescent patients with those of their parents to look for discrepancies between them.

## Methods

Thirteen consecutive patients with AIS returning to clinic for a follow-up visit, who had been wearing a TLSO brace for >3 months but <2 years, were recruited to participate. They were given a BrQ survey, and their parents were also given the same survey, with the wording changed to indicate that the questions were about the adolescent, and not the parent.

## Results

The correlation between the overall BrQ scores between patient and parent were high (r = 0.79). When the sub-scores were evaluated, the scores for Emotion, Self Esteem, Social Functioning, and Bodily Pain correlated most strongly. General Health, Physical Functioning, Vitality, and School Activity correlated the weakest. Parent scores were higher than patient scores 80% of the time.

## Conclusions

The BrQ survey showed that although patient and parent scores were similar, the parent gives the adolescent higher scores than they give themselves.
